# Coding, Decoding and Retrieving a Message Using DNA: An Experience from a Brazilian Center Research on DNA Data Storage

**DOI:** 10.3390/mi15040474

**Published:** 2024-03-30

**Authors:** Caio P. Gomes, André G. C. Martins, Sabrina E. Nunes, Bruno Ramos, Henrique R. Wisinewski, João L. M. S. Reis, Ariel P. Lima, Thiago Y. Aoyagi, Icaro Goncales, Danilo S. Maia, Ariane S. Tunussi, Marília S. Menossi, Sergio M. Pereira, Paula C. G. Turrini, João H. D. B. Gervasio, Bruno M. Verona, Natalia N. P. Cerize

**Affiliations:** 1 Bionanomanufacturing Center, Institute for Technological Research—IPT, Sao Paulo 05508-901, SP, Brazil; andremartins@ipt.br (A.G.C.M.); sabrinanunes@ipt.br (S.E.N.); henriquereis@ipt.br (H.R.W.); joaomaehara@ipt.br (J.L.M.S.R.); arielplima3@gmail.com (A.P.L.); thiagoaoyagi@ipt.br (T.Y.A.); icaro.gonc@gmail.com (I.G.); danilomaia@ipt.br (D.S.M.); aritunussi@ipt.br (A.S.T.); mariliam@ipt.br (M.S.M.); matias@ipt.br (S.M.P.J.); paulaturrini@ipt.br (P.C.G.T.); brunoverona@ipt.br (B.M.V.); ncerize@ipt.br (N.N.P.C.); 2 Microfluidic & Photoelectrocatalytic Engineering Group, Department of Chemical Engineering, FEI University Center, São Bernardo do Campo 09850-901, SP, Brazil; brunoramos@fei.edu.br

**Keywords:** DNA data storage, microfluidic device, DNA, synthesis, micromachine

## Abstract

DNA data storage based on synthetic oligonucleotides is a major attraction due to the possibility of storage over long periods. Nowadays, the quantity of data generated has been growing exponentially, and the storage capacity needs to keep pace with the growth caused by new technologies and globalization. Since DNA can hold a large amount of information with a high density and remains stable for hundreds of years, this technology offers a solution for current long-term data centers by reducing energy consumption and physical storage space. Currently, research institutes, technology companies, and universities are making significant efforts to meet the growing need for data storage. DNA data storage is a promising field, especially with the advancement of sequencing techniques and equipment, which now make it possible to read genomes (i.e., to retrieve the information) and process this data easily. To overcome the challenges associated with developing new technologies for DNA data storage, a message encoding and decoding exercise was conducted at a Brazilian research center. The exercise performed consisted of synthesizing oligonucleotides by the phosphoramidite route. An encoded message, using a coding scheme that adheres to DNA sequence constraints, was synthesized. After synthesis, the oligonucleotide was sequenced and decoded, and the information was fully recovered.

## 1. Introduction

Globalization has led to a substantial surge in data production, driven by rapid technological advancements and the widespread adoption of personal tech devices, thereby necessitating a continuously expanding storage capacity. With advances in technology, it is estimated that by 2040, the amount of data generated will reach 5 × 10^24^ bits [1]. Another relevant point in relation to data storage is the cost and handling time associated with storing information using current storage media. For example, information retention on a magnetic tape is estimated to be approximately 30 years or less [2].

Currently, there is a variety of physical means to store digital data, such as state solid drives, hard disks, etc. To produce and operate these devices, finite resources such as silicon and noble metals are consumed, even as electrical energy and physical space. With the growing population, these resources are becoming increasingly scarce.

The tremendous demand for data storage poses a risk of potential collapse as the power generation supply chain and the manufacturing capacity of this industry struggle to keep up with its growing pace [3,4]. New technologies are being studied to increase the data storage capacity to change this scenario. One technology showing significant promise is the storage of data in DNA molecules (also known as oligonucleotides or simply oligos). This technique, which involves replacing the traditional binary system (0, 1) used in computational data processing with a quaternary system (A, T, C, G), has the potential to revolutionize data storage.

Theoretically, due to DNA’s durability and very high storage density, adopting this system could not only increase data storage capacity but also create an archive that could last for thousands of years without the need for rewriting to keep the media readable. Through billions of years of natural selection, nature has identified DNA as the optimal polymer for storing and transmitting inherited information across generations. This data storage technique seems promising for imitating what nature already efficiently does. DNA stores information in a high-density manner, with each nucleus of a human cell having the potential for storing 800 MB in 3.2 billion bases pairs [5] and offers long stability, with the oldest mammoth genome being sequenced dates from Middle Pleistocene, one million years ago [6]. It is also noteworthy that one gram of DNA can store data in petabyte order, an extraordinary capacity, where a single test tube could easily replace a data storage center the size of a modern hypermarket [7]. In addition, the technology for copying the information stored within DNA, known as polymerase chain reaction (abbreviated PCR), is already commercially available. Therefore, this technology can offer gains in storage longevity and savings in resources such as materials, space, and electricity.

The massive synthesis of DNA is still a complex process, and the synthesis (writing) of DNA is expensive, especially long oligonucleotide sequences (>100 bases). But it is worth remembering that at the beginning of several technologies the initial use of their development tends to be costly, the first human genome for instance had a cost of billions of dollars in the mid-1990s and is currently below of thousand dollars [8].

Microfluidics technology is a rapidly expanding field within biotechnology, and it is revolutionizing biochemical processes with enhanced efficiency and sustainability. Our research explores a novel application of microfluidics in de novo DNA synthesis. This process involves creating DNA sequences from scratch without relying on existing DNA strands as templates [9]. This groundbreaking approach stands to transform the field of DNA synthesis. By integrating microfluidics, our technology not only significantly reduces waste production but also ensures cost-effective scaling of reactions. These advantages make it a highly appealing option for a wide range of scientific research, offering a new horizon in the exploration of genetic possibilities.

One of the most notable applications of microfluidics in biotechnology is its integration into the PCR process. Several research initiatives have successfully harnessed microfluidic systems to optimize the PCR process, achieving high-throughput DNA replication with minimal resource consumption. These advancements underscore the potential of microfluidics to streamline and enhance traditional biochemical methodologies [10,11]. The polymerase chain reaction (PCR) process has a limitation compared to de novo DNA synthesis since it requires a DNA strand to be used as a template for amplification. However, to store digital information in DNA, we rely on de novo DNA synthesis. Therefore, our research endeavors to explore a novel application of microfluidics—synthesizing DNA without relying on a pre-existing template. This groundbreaking approach harnesses the precision and control of microfluidic devices to synthesize DNA strands, providing a pathway to custom DNA sequences with specific characteristics.

Our work not only contributes to the expanding repertoire of microfluidics applications in biotechnology but also presents the versatility and potential of this technology to revolutionize genetic manipulation and synthesis, which can be applied to the biotechnology industry in addition to DNA data storage. Through the controlled environment of microfluidic systems, we aim to achieve unprecedented levels of accuracy and efficiency in DNA synthesis, setting a new standard for synthetic biology research and its practical applications. This could lead to significant advancements in genetic engineering, therapeutic developments, and the synthesis of bioactive molecules.

Research in DNA data storage has proliferated across various domains, penetrating even into cryptosecurity realms. Numerous studies have surfaced, exploring diverse methodologies harnessing DNA encoding to generate cryptographic keys, fortifying the security of stored information in digital archives or within physical DNA structures. Colocar referencias da pasta artigo [12].

**Figure 1:**
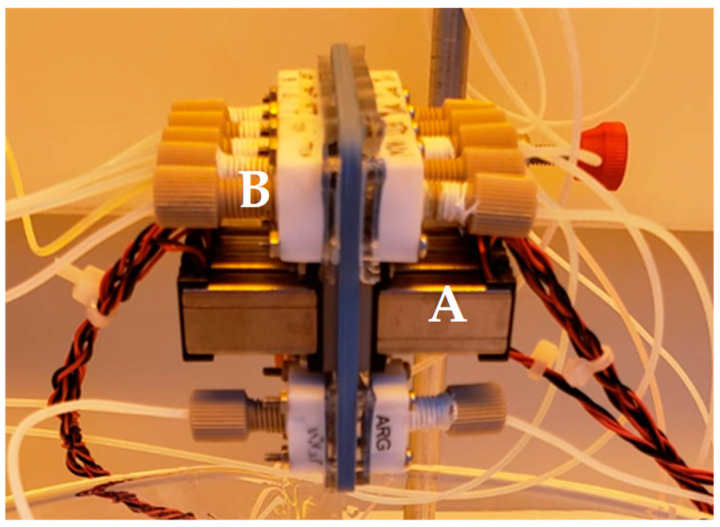
Device for fluids management in LTCC. A—Microvalves. B—Peek and Teflon connections.

**Figure 2:**
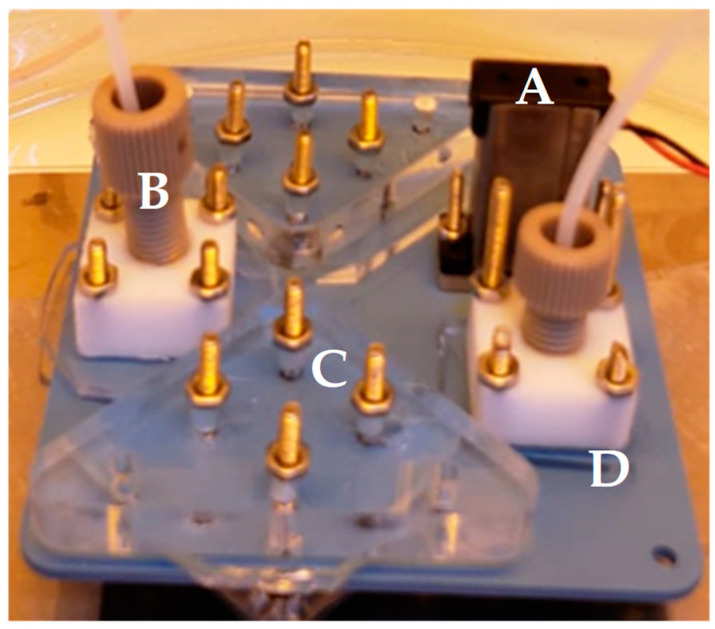
Device module containing DNA reaction sites. A—Microvalves. B—Peek and Teflon connections. C—Reaction site. D—LTCC Device.

Herein, we present a proof of concept for a DNA synthesizing process using a microdevice that can be parallelized. Parts of the development of this microdevice are currently submitted for patent protection, and in-depth details will not be covered in this work. Our device consists of two modules: the first is a valve system that manages liquid flow (see Figure 1), and the second contains the reaction sites for DNA synthesis (see Figure 2). We have integrated electronic circuits to control the insertion of fluids, utilizing pressure and time as variables. Each reagent injection consumes approximately 90 microliters, sufficient to produce 10^13^ copies of DNA at each reaction site. The novelty lies in utilizing LTCC (Low-Temperature Co-fired Ceramic) as a substrate, enabling minimal reagent consumption and shorter synthesis times compared to traditional commercial equipment. Another intriguing prospect involves embedding electronics within LTCC. The most commonly employed method for oligonucleotide synthesis is the phosphoramidite route, which typically achieves a writing speed of 1 base every 4–6 min [5], while our device boasts a significantly faster writing speed of around 2 min per base.

## 2. State of the Art—First Experiment, the Proof of Concept

The first experiment at our center aimed to evaluate the feasibility of developing both a project and the underlying technology. The conceptualization and development of this technology relies on professionals with expertise in diverse fields, including engineering, chemistry, molecular biology and information technology. We embarked on an experiment to encode, synthesize, and decode a message, employing coding and decoding algorithms documented in existing literature. We named our project ‘Prometheus’.

**Figure 3:**
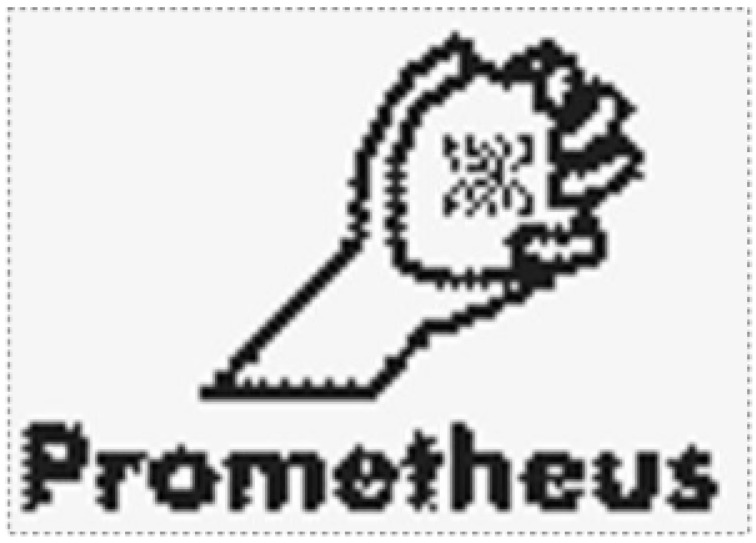
Image of the project logo, encoded and decoded in the first experiment.

For this experiment, we encoded the project logo using an algorithm developed by Organick et al., published in 2018 [13]. The encoding process produced 23 oligonucleotides, each with 192 bases in length, from an original image file of 536 bytes (see Figure 3). These oligonucleotides include sequences for PCR amplification primers and unique identifiers at each end, yielding a net payload of 95 bases per oligonucleotide.

The oligonucleotides were synthesized through commercial means. Subsequently, each oligonucleotide was replicated using PCR and then sequenced using the Nanopore platform. The sequencing produced reads that were successfully aligned, and the files were successfully retrieved and decoded.

Strands data are susceptible to errors during synthesis and sequencing. These errors often correlate with specific DNA subsequence patterns, such as homopolymers with long run lengths and unbalanced GC content. To minimize errors, most mapping methods aim to circumvent these problematic subsequences.

A complementary strategy widely employed in DNA data storage to deal with these errors is using error-correcting codes (ECC), such as Reed-Solomon [14] and LDPC, which are well-established methods in the communication field. These codes insert redundancy into the encoded message (therefore increasing the amount of DNA to be synthesized and sequenced), allowing the recovery of corrupted data.

In summary, the encoding process performs:

- File segmentation and segment addressing;
- Mapping bits to DNA bases;
- Insertion of redundant data (for posterior error correction);
- Appending primers (for PCR amplification purposes).

### 2.1. Experiment Design

In this experiment, we encoded a small, 38-byte text file. A small file was intentionally selected to minimize the costs associated with synthesis and sequencing for this test. A text file is particularly suitable for small-scale experiments due to its straightforward interpretation, though any file type could be utilized.

We adopted the mapping method proposed by Blawat [15], featuring a reasonable storage rate of 1.6 bits/nt. This method allows for the control of undesired DNA subsequences through degenerate mapping, providing multiple coding options for each chunk of input bits. We ensured the absence of homopolymers with run lengths of four or more, avoided primers and the correspondent self-reverse complements to prevent readout errors during sequencing, and controlled the local GC content in every subsequence of 15 nucleotides to approximately 50%. A cost function automatically selects the coding option, considering the mentioned constraints.

For error correction coding (ECC), we utilized the Reed-Solomon (RS) code, inserting two redundant symbols in each oligonucleotide (oligo) to correct any single erroneous symbol per oligo. We used an RS symbol size of 8 bits (one byte), a common practice that accommodates coded messages with up to 255 RS symbols (2^8^ − 1), suitable for this experiment. After the Blawat mapping, the RS code was directly applied to the DNA sequences. Considering every four bases mapped to one RS symbol (equivalent to 2 bits per base), we added eight bases of logical redundancy to each oligo for error correction. Although this approach theoretically allows correcting up to four bases with errors within the same RS symbol, in practice, it ensures the correction of a single erroneous base per oligo (excluding primer regions, which are beyond the scope of code protection).

**Figure 4:**
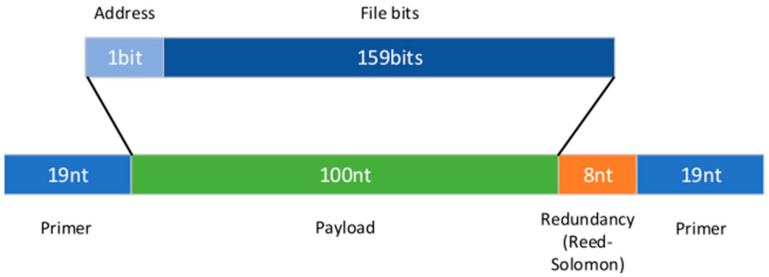
Oligo content structure.

We determined the maximum oligo length to be 150 nucleotides (nt), which is appropriate for the synthesis technologies used in this experiment (Figure 4). Given the length of each primer of 19 nt (M13 forward and M13 reverse) and the allocation of 8 nt for logical redundancy, the available DNA length for data and address—referred to as the payload—is 104 nt (150-38-8). The Blawat mapping requires the payload size to be a multiple of 5 nt, and the RS code requires it to be a multiple of 4 nt, thus necessitating a payload size that is a multiple of 20 nt. Consequently, the most suitable payload size for this experiment was 100 nt, resulting in oligonucleotides of 146 nt in length (Table 1).

Given that the payload of each oligo contains 160 bits (1.6 × 100) and the input file totals 304 bits (38 × 8), we divided the file into two segments for storage in two oligos, using a single address bit. The file did not fully occupy the available space in the two payload segments, prompting us to append fourteen ‘0’ bits at the end to fill the gap. Finally, we exported the two DNA sequences containing the coded information into a FASTA file for synthesis.

Experimental data:
Encoded file: 38-byte text file containing the following sentence:
IPT e Lenovo, uma parceria de sucesso!
(IPT and Lenovo, a successful partnership!)
Primers segments in the synthesized strand:
CACGACGTTGTAAAACGAC (forward M13)
GGGTCATAGCTGTTTCCTG (reverse M13—reverse-complementary)
Output oligos: 2 oligos of size 146 nt.
FASTA file content (oligo basewise content):
>Ep1
CACGACGTTGTAAAACGACAGACAGGAGAAGCGTACTATATAAGGCCACAGACGATAAGGTGCTATCCGGTAGCATGCTGCACGACTATATCGTGTACGGTCACGCTATATCGCATCACGGGACGCCGGGTCATAGCTGTTTCCTG
>Ep2
CACGACGTTGTAAAACGACTCGTGGCAGATCAGTCCATAGCCGTCCAGACAAGAACAGTACGGCCAAGAACATATCGTCCCAGATCCGACCATATCCTCTCTGATACGCCATATAACACAGAGGGATGGGTCATAGCTGTTTCCTG

In this experiment, we utilized state-of-the-art methods for encoding and decoding to assess error correction capabilities and the efficacy of mapping schemes in adhering to DNA constraints.

During the design phase, we prioritized the discussion of self-reverse complementarity (secondary structures)—a DNA constraint often overlooked in the literature. Secondary structures pose potential challenges during the amplification stages between synthesis and sequencing. Further in this text, we will refer to self-reverse complementarity as ‘self-RC.’

Two main concerns arise regarding the importance of avoiding self-RC segments in DNA strands:

- Completely avoiding self-RC segments within encoded oligonucleotides is nearly impossible, as generating self-RC segments of length two is almost inevitable. A key question emerges: How can we determine the critical size or other characteristics of self-RC segments that contribute to errors during synthesis, amplification, and sequencing?
- With the addition of this DNA constraint, the coding process becomes more restrictive. Depending on the chosen mapping scheme, it may be challenging to avoid every constraint. Thus, it is crucial to identify which constraints are most significant. In essence, we aim to discern which DNA subsequence patterns are most likely to cause errors during synthesis, amplification, and sequencing.

### 2.2. Device Manufacturing

The fabrication of the synthesis devices was based on microfabrication and microfluidics techniques. This system reduces reagent consumption, facilitates controlled mixing of materials, and increases the efficiency of material or mixture production compared to micrometric-scale devices.

In this project, we utilized miniaturized devices for oligonucleotide synthesis. The chips were manufactured from low-temperature co-fired ceramics. Initially, AutoCad 2021 software was used to prepare the layout of the ceramic layers. The device was then manufactured by transferring the layouts to ceramic sheets using laser cutting with an LPKF Protolaser U3.

Following the laser cutting, the ceramic layers were aligned according to the designed layout. The subsequent step, lamination, involved adhering the ceramic sheets under pressure and slight heat, following the manufacturer’s protocol: a gluing step at 70 °C for ten minutes, followed by pressing the sheets at 70 °C under 3 Torr pressure. Sintering, the final manufacturing stage of the chip, involved placing the ceramic in an oven set to a specific sintering level.

**Figure 5:**
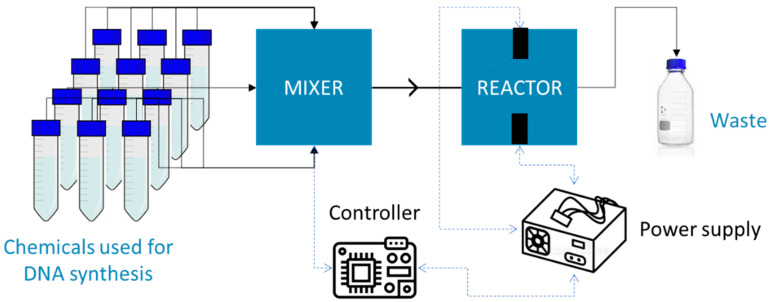
Schematic representation of the manufactured device.

The system comprises two connected LTCC (Low-Temperature Co-fired Ceramic) chips, as depicted in Figure 5. The first chip serves as the reagent selector, tasked with mixing reagents and controlling their delivery to the reactor. It features 11 inputs and a single output, equipped with 12 valves (one for each terminal, including the output). The output from the mixer is then directed to the second chip, which houses two synthesis chambers. Valve actuation hardware connects to the mixer, ensuring precise control over the process.

The valve used in the mixer is the Burkert 6712, which directly handles the reagents. Valves are actuated with 24 V, then we used a benchtop power supply with the following specifications: maximum output 0 to 35 V/1.4 or 0 to 60 V/0.8 Â. It boasts a fast switching time (2 milliseconds) and is expected to have no carry-over volume or dead-volume, thereby enabling controlled reagent consumption and mixing. 

The synthesis reactor consists of two reaction cradles arranged in parallel. This simple structure facilitates the parallelization of the synthesis process, which is aimed at exploring and developing synthesis methodologies.

The electronics were designed to synchronize the valve control module with both the source and the current module. For this purpose, I^2^C communication was employed, a synchronous master/slave protocol that utilizes two communication pins: SDA and SCL. The valve control module, which regulates the flow of nucleotides through the synthesizer module, receives commands via USB. In this setup, it functions as the master, overseeing the operations of the source module.

### 2.3. DNA Synthesis

The oligonucleotides comprising the coded information were synthesized by the phosphoramidite route method, which is widely used in commercial equipment and oligonucleotide synthesis companies for use in the biological field. The reagents used in the experiment were supplied by Sigma Aldrich (San Luis, MO, USA) The amount of reagents consumed and reaction times for each synthesis cycle are described in Table 2. The execution time is approximately 120 s per base.

After synthesis, the oligonucleotides were cleaved and deprotected from the solid support using aqueous ammonia solution. After, the oligonucleotides were purified using a reverse phase oligonucleotide purification cartridge (RP-OPC) Microamp II from Biosearch according to manufacturing instructions.

### 2.4. Sequencing

**Figure 6:**
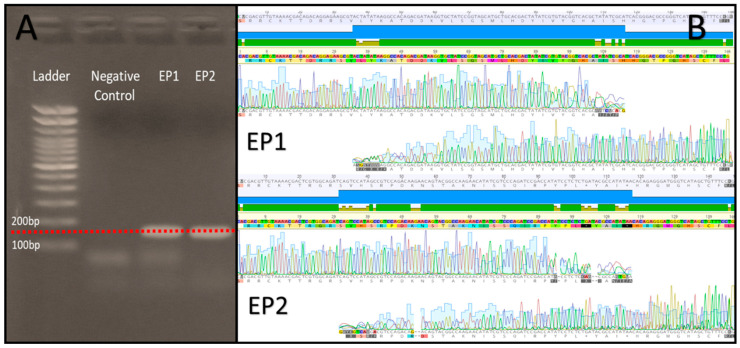
(**A**)—Fragment gel electrophoresis. (**B**)—sequencing result.

In this experiment, we employed the Sanger sequencing methodology to assess the base-to-base quality of the synthesis performed in our device. The produced oligonucleotides have two coupling sites for M13 primers (sequences). After purification, the oligonucleotides (ssDNA) were converted to double-stranded DNA (dsDNA) through PCR, using GoTaq Green Master Mix (Promega, Madison, WI, USA), according to the manufacturer’s instructions. The PCR products underwent electrophoresis to evaluate the fragments, as shown in Figure 6A; subsequently, the fragment was cut from the gel and purified using the Qiagen Gel Extraction Kit (Qiagen, Hilden, Germany), following the manufacturer’s instructions. After purification, the sequencing reaction was performed using the Big Dye Kit v3 (Applied biosystems, Waltham, MA, USA)) according to the manufacturer’s instructions. The sequencing reaction was read in the 3500 XL Applied Biosystems equipment using the POP7 polymer (Applied biosystems, Waltham, MA, USA), and the generated electropherograms were aligned with the respective reference sequences and analyzed with the Geneious software version R10 (Figure 6B).

We can observe in the image above that there is the fragment synthesized after PCR (Figure 6A) and the sequencing confirms that the synthesized fragment is correct, being identical to the expected reference sequence (Figure 6B).

## 3. Forecasting Trends, Market (Technical and Economic Feasibility) and Technology Challenges

The development of DNA data storage technology marks a significant advancement in the field of information science, a response to the escalating demand for data storage media and capacity. DNA emerges as a highly efficient and durable medium for data storage [6]. It boasts superior compression, volumetric density, longevity, and energy efficiency compared to conventional digital storage mediums [16]. However, the successful integration of DNA in data storage systems necessitates careful consideration of metadata integration, bio-cybersecurity measures, standardization of coding and decoding processes, and the inclusion of distinct markers for future data retrieval and recognition [5]. These factors are pivotal in harnessing DNA’s full potential as a data storage solution, ensuring that its capabilities are effectively leveraged for future technological advancements.

In order for this technology to transition into real-life applications, a multi-disciplinary approach is necessary that can contribute to promoting integration with current digital infrastructures, which demands the development of sophisticated interfaces and coding/decoding software [16]. These tools are essential for effectively translating digital data into DNA sequences and vice versa, enabling practical and efficient use of this innovative storage method [4]. Initial adoption is likely to be in specialized sectors that benefit from long-term, high-density storage, such as archival repositories in libraries, scientific databases, and cultural institutions [17]. Furthermore, collaboration with tech industry leaders and innovative startups will be vital for overcoming technical and economic barriers, particularly in scaling up DNA synthesis and improving retrieval speeds. Standardization of protocols and practices is another key aspect, ensuring reliability and scalability across various applications [18]. With focused research and development, coupled with industrial partnerships, DNA data storage could evolve from a niche scientific concept to a practical, widely used solution for the ever-growing global data storage needs [5].

Several research groups have demonstrated DNA’s data storage capacity and shown promising results, yet they have also highlighted key challenges that need to be addressed [19]. The high costs and low speed associated with DNA synthesis and data retrieval are significant obstacles that must be overcome to make DNA data storage a practical and marketable technology [18]. Despite these challenges, the field has witnessed considerable advancements, with the development of new encoding, decoding, and storage strategies, as well as improvements in DNA synthesis and sequencing equipment [17].

In the field of DNA data storage, current challenges include the high cost and environmental impact of DNA synthesis, particularly with phosphoramidite synthesis [18]. The process of synthesizing DNA using the phosphoramidite method is not only expensive, costing about $0.10 to $0.30 per base (which amounts to a staggering $800 million to $2 billion for 1 GB of data), but it also poses environmental concerns due to the chemicals used. This cost is significantly higher than traditional hard disk drives (HDDs), which are priced at around $0.30 per GB [4]. While future reductions in cost are anticipated, a significant breakthrough is yet to be achieved. As an alternative, enzyme-based DNA synthesis is being explored for its potential to lower costs and reduce environmental impact [20]. Moreover, advancements in sequencing technologies like next-generation sequencing (NGS) and Oxford Nanopore Technologies (ONT) nanopore sequencing are making sequencing faster, more accurate, and less error-prone [21]. These advancements are crucial for improving the efficiency and reliability of DNA data storage, but overcoming the cost and environmental challenges of DNA synthesis remains a key priority for the large-scale adoption of this technology [4,22]. Addressing these issues through innovative technological solutions is essential for the realization of DNA data storage as a practical and commercially viable alternative to conventional data storage methods.

Despite these hurdles, the field of DNA data storage has made significant strides. Advances in molecular biology, nanotechnology, novel polymers, electronics, and automation are collaboratively pushing the boundaries of what is possible, bringing DNA data storage closer to practical application and market viability [4]. The synthesis of DNA for data storage is being improved through the application of molecular biology and enzymes for efficient DNA synthesis and read-out [20], polymer technology [23], electronics [24], nanotechnology [18], and automation [19].

In conclusion, we were able to successfully encode different types of binary data (text and images) in DNA using our own codec system, synthesize the DNA using our engineered writing machine, and recover the information using DNA commercial sequencing technologies. Our current device is a proof of concept and does not meet the actual demand for oligonucleotide production required for data storage purposes. Our team is developing a microfluidic electrochemical system that promises to parallelize the synthesis process.

## Figures and Tables

**Table 1:** Characteristics of the oligos encoded for the experiment.

GC content at every 15 nt segments	Mean: 0.5079 Std Dev: 0.0884
Homopolymer counting	run-length 2: 30 run-length 3: 7 run-length 4: 2
Undesired motifs (primers and reverse-complementars)	CACGACGTTGTAAAACGAC:2 GTCGTTTTACAACGTCGTG: 0 GGGTCATAGCTGTTTCCTG: 2 CAGGAAACAGCTATGACCC:0
Counts of self-reverse complementary segments (search based only on the size of the reverse-complementary segment)	run-length 3: 220 run-length 4: 60 run-length 5: 16 run-length 7: 1
Longest self-reverse complementary pair: segments (71, 78) and (72, 79) from the first oligo.	(forward sequence) GGTGCTATCCGGTAGCATGCTGCACGACTATATC |||||||| CTATATCAGCACGTCGTACGATGGCCTATCGTGG (reverse sequence)

**Table 2:** Reagents, time and volume used in the cycle synthesis.

Cycle (Reagent)	Reaction Time (Seconds)	Amount (Microliters)
Wash (Acetonitrile and Argon)	none	180
TCA (Trichloroacetic acid in Dichloromethane)	30	90
Wash (Acetonitrile and Argon)	none	180
Protected DMT base adiction (A,T,C,G) in Acetonitrile anhydrous (0.1 M)	30	90
Wash (Acetonitrile and Argon)	none	180
CAP MIX (CAP B-1-methylimidazole 16% in THF)—(CAP A—Tetrahydrofurane/Lutidine/Acetic Anhydride 8/1/1)	30	90
Wash (Acetonitrile and Argon)	none	180
Oxidizer (Tetrahyrofurane/Water/Pyridine/Iodine 77/2/21/2.54)	30	90

## Data Availability

The original contributions presented in the study are included in the article, further inquiries can be directed to the corresponding authors.
